# Reducing the Intensity and Volume of Interval Training Diminishes Cardiovascular Adaptation but Not Mitochondrial Biogenesis in Overweight/Obese Men

**DOI:** 10.1371/journal.pone.0068091

**Published:** 2013-07-05

**Authors:** J. Colin Boyd, Craig A. Simpson, Mary E. Jung, Brendon J. Gurd

**Affiliations:** 1 School of Kinesiology and Health Studies, Queen’s University, Kingston, Ontario, Canada; 2 Department of Emergency Medicine, Queen’s University, Kingston, Ontario, Canada; 3 Faculty of Health and Social Development, University of British Columbia at Okanagan, Kelowna, British Columbia, Canada; University of Bath, United Kingdom

## Abstract

**Objective:**

The purpose of this research was to determine if the adaptations to high intensity interval training (HIT) are mitigated when both intensity and training volume (i.e. exercise energy expenditure) are reduced.

**Methods:**

19 overweight/obese, sedentary males (Age: 22.7±3.9 yrs, Body Mass Index: 31.4±2.6 kg/m^2^, Waist Circumference: 106.5±6.6 cm) performed 9 sessions of interval training using a 1-min on, 1-min off protocol on a cycle ergometer over three weeks at either 70% (LO) or 100% (HI) peak work rate.

**Results:**

Cytochrome oxidase I protein content, cytochrome oxidase IV protein content, and citrate synthase maximal activity all demonstrated similar increases between groups with a significant effect of training for each. β-hydroxyacyl-CoA dehydrogenase maximal activity tended to increase with training but did not reach statistical significance (p = 0.07). Peroxisome proliferator-activated receptor gamma coactivator-1α and silent mating type information regulator 2 homolog 1 protein contents also increased significantly (p = 0.047), while AMP-activated protein kinase protein content decreased following the intervention (p = 0.019). VO_2_peak increased by 11.0±7.4% and 27.7±4.4% in the LO and HI groups respectively with significant effects of both training (p<0.001) and interaction (p = 0.027). Exercise performance improved by 8.6±7.6% in the LO group and 14.1±4.3% in the HI group with a significant effect of training and a significant difference in the improvement between groups. There were no differences in perceived enjoyment or self-efficacy between groups despite significantly lower affect scores during training in the HI group.

**Conclusions:**

While improvements in aerobic capacity and exercise performance were different between groups, changes in oxidative capacity were similar despite reductions in both training intensity and volume.

## Introduction

High intensity interval training (HIT), a training modality that alternates between brief repeated bursts of intense exercise and periods of active rest, improves several clinically relevant outcomes. Specifically, in both healthy and clinical populations, HIT improves VO_2_peak, exercise performance, cardiovascular function, and markers of oxidative capacity in skeletal muscle [1]. HIT has also recently been reported to reduce systemic inflammation in both coronary intervention patients [2] and individuals with metabolic syndrome [3].

These physiological benefits can be achieved in less time and with less total exercise energy expenditure (i.e. exercise volume) than traditional endurance training (ET) [4], [5]. However, while some evidence may suggest it is more enjoyable than ET [6], HIT is typically associated with near maximal or supramaximal intensities. These intensities represent a potential threat to the adoption of HIT by the general population as high intensity exercise is typically associated with negative feelings and poor exercise adherence [7], particularly in overweight/obese populations [8]. The safety of higher intensity protocols (i.e., supramaximal intervals) for populations with cardiovascular risk or disease has also been questioned [9].

Accordingly, there is a need for studies designed to determine whether reductions in the intensity of HIT that also result in decreases in exercise volume (i.e. decreases in the energy expenditure associated with exercise), result in a loss of physiological adaptation such as improved aerobic capacity, skeletal muscle oxidative capacity, cardiovascular function, inflammatory response, etc. These studies are important as low intensity/low volume HIT would be expected to elicit less negative feelings and safety concerns typically associated with high intensity exercise [1], [9]. While interval duration has been examined in cardiac patients [10] and both interval duration [11] and training frequency [12] have been studied in young healthy adults, the impact of reducing both interval intensity and training volume is unknown. While recent evidence suggests that reduced-exertion HIT can result in important physiological adaptations [13], the question of how these improvements compare with those elicited by high intensity, high volume training programs remain.

Therefore, the purpose of this study was to compare the physiological adaptations resulting from regular performance of a high intensity/high volume (HI) interval training protocol to adaptations resulting from a low intensity/low volume (LO) interval training protocol. Changes in skeletal muscle oxidative capacity, aerobic capacity, exercise performance, peak O_2_ pulse, inflammation status, and perceived tolerability where examined in overweight/obese individuals. Specifically, we examined the above variables before and after 3 weeks of interval training using a 1-minute on, 1-minute off protocol (adapted from Hood et al. [14]) at an interval intensity of either 100% (HI) or 70% (LO) of peak aerobic power. We hypothesized that increases in all measures of muscle oxidative capacity and aerobic function would be greater following HI than LO training. We also hypothesized that improvements in inflammatory status would be greatest in the HI group but that overall perceived tolerability would be highest following LO.

## Methods and Procedures

### Participants

Nineteen overweight/obese, sedentary males volunteered to participate in this study (participant characteristics are presented in Table 1). All participants were between 18–35 years old, reported participating in less than 1 hour per week of aerobic exercise (jogging, cycling, etc.) at enrollment, and had a waist circumference greater than 94 cm [15]. Participants were matched on pre-test waist circumference and VO_2_peak before being stratified into two groups completing 3 weeks of cycling training utilizing repeated intervals of either high intensity/high volume (100% peak aerobic power; HI) or low intensity/low volume (70% peak aerobic power; LO). Both groups performed the same number of intervals during each training session such that the total duration of each training session was matched.

**Table 1:** Participant Characteristics.

	LO		HI	
	Pre	Post	Pre	Post
**N**	10	–	9	–
**Age (yrs.)**	22.7±4.3	–	22.7±3.8	–
**Height (cm)**	184±8	–	180±7	–
**Body mass (kg)**	105±14	103±15	102±12	102±11
**BMI (kg/m^2^)**	30.7±3.0	30.2±3.0	32.3±2.1	32.2±1.9
**Waist circumference (cm)**	105±8	103±8	108±5	107±6
**Abs. VO_2_peak (ml/min)**	3619±954	3892±663†	3607±594	4489±486† §
**Rel. VO_2_peak (ml/kg/min)**	35.8±8.2	38.6±6.5†	35.4±5.7	44.7±4.9† §
**Peak power (W)**	293±39	313±47†	308±49	336±49†
**HRpeak (bpm)**	189±11	188±8†	197±8	191±10†
**Peak O_2_ pulse (mL/min/bpm)**	20.9±4.4	22.7±4.9†	18.8±3.2	24.3±3.6† §
**Time to 500 kcal (s)**	2481±560	2277±588†	2365±599	2034±533†
**Glucose (mmol/L)**	4.7±0.2	4.7±0.2	4.8±0.4	4.7±0.4
**Insulin (µIU)**	10.0±2.5	10.5±3.2	12.1±5.5	10.9±4.2
**HOMA-IR**	2.1±0.6	2.1±0.6	2.5±1.1	2.3±0.9
**Training HR (bpm)**	141±17	–	166±12‡	–
**Interval WR (W)**	206±27	–	308±48‡	–

Values are mean ± SD. yrs, years; cm, centimetres; kg, kilograms; BMI, body mass index; m, metres; mmol/L, millimoles per litre; µIU, micro international units; HOMA-IR, homeostatic model assessment of insulin resistance; W, watts; s, seconds; HRpeak, maximal heart rate from VO_2_peak test; bpm, beats per minute; Training HR, average heart rate from first training session. Interval WR, average power produced during intervals from first training session.

† Significant (p<0.05) effect of training.

‡ Significantly different (p<0.05) from LO.

§ Significant (p<0.05) interaction between groups.

### Ethics Statement

This study protocol conformed to the Ethical Guidelines outlined by the Declaration of Helsinki and was approved by the Health Sciences Human Research Ethics Board at Queen’s University. All participants provided informed written consent prior to participation in the study.

### Baseline Testing

Participants arrived for the first laboratory visit in the morning following an overnight fast (≥8 h). Resting blood samples were collected by venipuncture from an antecubital vein in sterile tubes (BD Vacutainer, Franklin Lakes, NJ) with and without EDTA as an anticoagulant. Plasma was separated by centrifugation at 3500 RPM for 10 minutes at 4°C while serum was separated by centrifugation at 3500 RPM for 15 minutes at 4°C. Samples were stored at −80°C until analysis. Following blood sampling, participants were fed a standardized breakfast consisting of a bagel (1 g Fat, 6 g Protein, 39 g Carb, 190 kcal) with cream cheese spread (18 g Fat, 4 g Protein, 2 g Carb, 200 kcal) and a juice box (0 g Fat, 2 g Protein, 26 g Carb, 110 kcal). Participants then remained seated in a chair for 1 hour before a resting muscle biopsy was obtained using the Bergstrom needle biopsy technique [16]. Biopsies were performed under sterile conditions with local anesthesia (2% lidocaine) using a custom modified Bergstrom biopsy needle and manually-applied suction. Muscle tissue was immediately blotted, snap-frozen in liquid nitrogen, and stored at −80°C until analysis.

Forty-eight hours following the muscle biopsy, participants returned to the lab for a VO_2_peak incremental ramp test to exhaustion on a Monark Ergomedic 874E stationary ergometer (Vansbro, Sweden). The VO_2_peak ramp protocol consists of a five minute loadless warm-up followed by a step increase to 70 W for one minute and subsequent increases in work rate of 21 W⋅min^−1^ to volitional exhaustion or the failure of the participant to maintain a cadence of 60 RPM. Gas exchange and heart rate were measured with a metabolic cart (Moxus AEI Technologies, Pittsburgh, PA) calibrated before each test using a two-point gas calibration (known gas and atmospheric air) and a 3L syringe for volume calibration. Relative VO_2_peak, absolute VO_2_peak and HRpeak were selected as the highest value of continuous 30 s averages for each measure during the protocol. Peak O_2_ pulse was calculated as absolute VO_2_peak divided by HRpeak from the incremental ramp protocol.

Twenty-four hours following the incremental ramp test, participants completed a 500 kcal time to completion trial at a self-selected cadence as quickly as possible against a load expected to elicit 50% VO_2_peak at 60 RPM [17]. Total external work (expressed in kcal) was calculated by the cycle ergometer based on load (kiloponds) and cycle speed (rpm). Participants were given no temporal, verbal, or physiological feedback and were only aware of how many calories had been expended. Exercise duration was recorded for each test.

### Training Protocol

Training was performed three times per week for three weeks with the number of intervals completed per training session increasing each week, starting at 8 intervals per session for Week 1, 9 per session in Week 2, and 10 per session in Week 3. Each training session consisted of a five minute loadless warm-up, followed by repeated 60 s intervals at either 100% (HI) or 70% (LO) of baseline testing maximal aerobic power while maintaining a cadence of 80 RPM. Each interval was followed by 60 s of loadless cycling at 80 RPM and all training sessions ended with a five minute loadless cool down period.

To assess perceived affect (i.e., valence feelings of pleasure – displeasure), the Feeling Scale (FS) was also administered during each participant’s first training session of each week of training. This single-item, validated 11-point Likert bipolar FS ranged from −5 (Very Bad) to +5 (Very Good).

### Post-training Measures

Post-training tests were conducted in an identical manner as the baseline measures. Fasted blood and a resting muscle biopsy were sampled 72 h following the final training session. 48 h after the muscle biopsy, participants performed an incremental VO_2_peak ramp protocol, then a 500 kcal time to completion trial 24 h later. Participants were also asked about how much they enjoyed the exercise they engaged in as well as their confidence to continue to engage in it. Perceived enjoyment was assessed by the question “How enjoyable would it be for you to do high intensity interval training 3 days per week?” Responses were recorded on a scale of 1 (not enjoyable at all) to 7 (extremely enjoyable). Scheduling self-efficacy was assessed using a single item measure of confidence “How confident are you that you could schedule interval training sessions three times per week?” and task self-efficacy was assessed using the single item measure “How confident are you that you would complete interval training sessions three times per week?” Both self-efficacy questions utilized a 10-point Likert scale ranging from 1 (not confident at all) to 10 (completely confident). Intentions to implement high intensity exercise following completion of the study was assessed by asking participants “at the completion of this study, I intend to add hard, or very hard exercise of at least 30 minutes to my leisure time physical activity”, with items being “at least once per week”, “three times per week”, and “five times per week”. Intention to implement questions utilized a 7-point Likert scale ranging from 1 (strongly disagree) to 7 (strongly agree).

### Western Blot Analysis

30–50 mg of frozen muscle tissue was homogenized in pre-chilled lysis buffer supplemented with Halt Protease Inhibitor Cocktail (100X, Thermo Fisher Scientific, Rockford, IL). Protein concentrations were determined by protein assay (Pierce, Rockford, IL) and equal amounts of total protein were loaded and separated by SDS-PAGE using an 8.0% (PGC-1α, AMPKα), 10.0% (COX I, COX IV), or 12.0% (SIRT1) polyacrylamide gel before subsequent transfer to a polyvinylidene difluoride membrane. Commercially available antibodies were used for the detection of PGC-1α (Calbiochem, San Diego, CA), AMPKα, GAPDH (Millipore, Temecula, CA), COX I, COX IV (Cell Signalling, Danvers, MA), and SIRT1 (Abcam, Cambridge, MA). Proteins were visualized by chemiluminescence detection according to the manufacturer’s instructions (Millipore, Billerica, MA). Blots were imaged using the FluorChem Cell Biosciences imaging system and quantified using AlphaView technology. Equal protein loading for all Western blots were confirmed using GAPDH.

### Citrate Synthase and β-Hydroxyacyl-CoA Dehydrogenase Activity

An additional portion of muscle (∼20 mg) was homogenized by hand for 30 s in glass hand homogenizers on ice and used to determine maximal citrate synthase (CS) and β-hydroxyacyl-CoA dehydrogenase (βHAD) activity. Total CS activity was measured spectrophotometrically at 37°C using the colormetric agent DTNB at 412 nm. βHAD activity was determined at 37°C by determining the rate of disappearance of NADH at 340 nm.

### Blood Analysis

Fasted blood glucose was determined via a hexokinase reaction assay performed at the Kingston General Hospital (Kingston, Ontario). Fasted insulin levels were determined with a commercially available enzyme-linked immunoabsorbent assay (ELISA) kit (ALPCO Diagnostics, Salem, NH). All samples were run in duplicate, with the CV being <10% for all values. Insulin sensitivity was estimated using homeostatic model assessment – insulin resistance (HOMA-IR) with the equation:

HOMA-IR = [fasting insulin (µIU/mL)×fasting blood glucose (mmol/L)]/22.5.

Plasma interleukin-6 (IL-6), tumor necrosis factor alpha (TNFα), and adiponectin were determined using commercially available high sensitivity ELISA kits (R & D Systems, Minneapolis, MN). All samples from individual participants were tested in duplicate on the same assay plate. Repeat analysis was performed on duplicates that varied by more than 15% and the average of all repeats was used for analyses. Values are reported in pg/mL (IL-6, TNFα) and ng/mL (adiponectin).

### Statistics

A two-way, repeated measure ANOVA was used to compare the effects of time (training status) and interval intensity (group). Data analysis was completed with GraphPad Prism v 5.01 (GraphPad Software, Inc., La Jolla, CA). Statistical significance was accepted at p<0.05 unless otherwise noted.

## Results

### Muscle Oxidative Capacity

**Figure 1:**
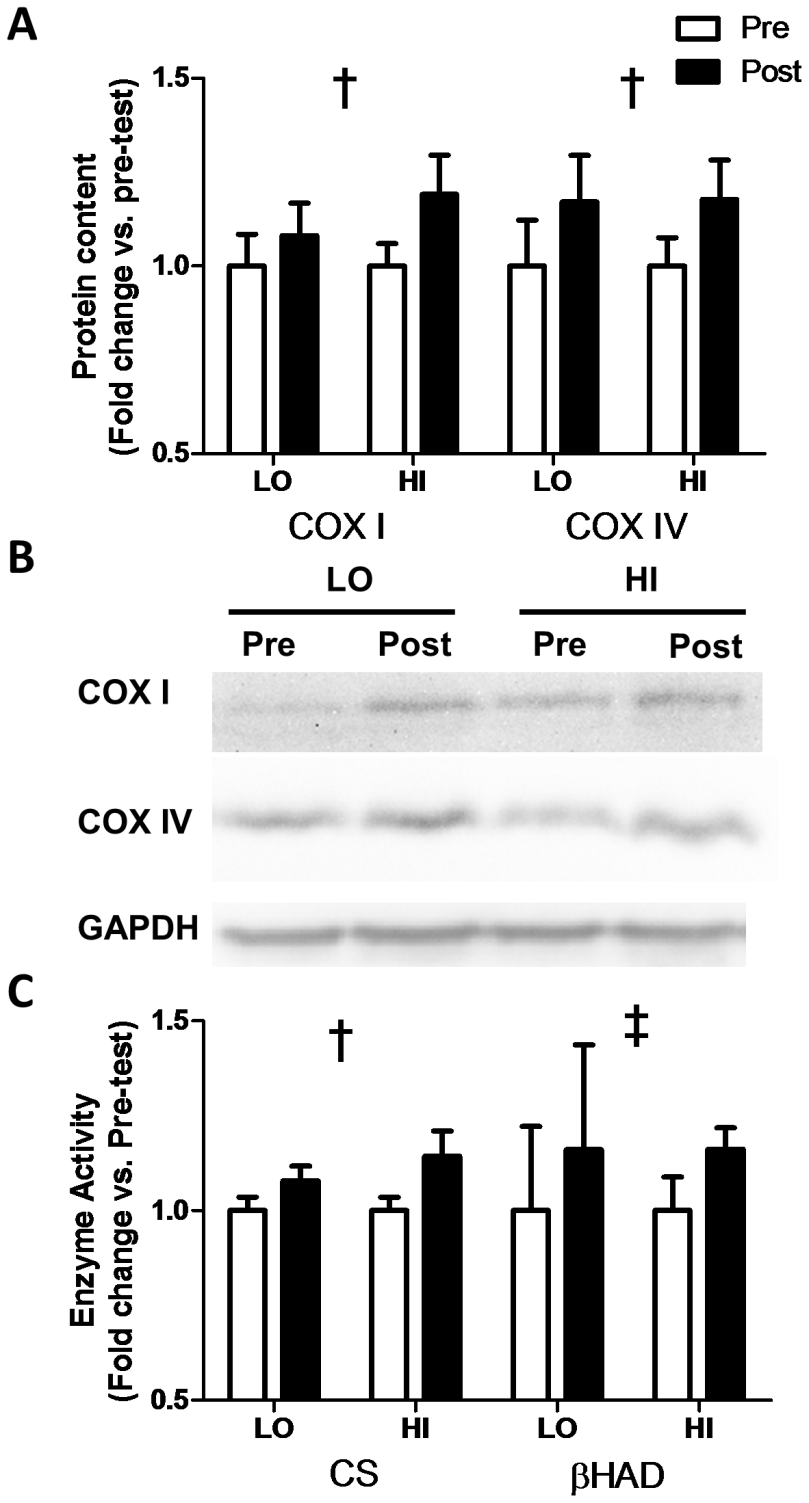
Effect of HI and LO on markers of skeletal muscle oxidative capacity. Changes in protein content of COX I and COX IV (A) and the maximal enzyme activities of citrate synthase (CS) and β-hydroxyacyl-CoA dehydrogenase (βHAD) (C). Representative western blots, including loading controls, are also shown (B). † Significant (p<0.05) effect of training. ‡ Non-significant (p = 0.07) effect of training.

A main effect of training (p<0.01; Figure 1A) was observed for both COX I (LO, Pre-test: 1±0.09 Arbitrary Units (AU), Post-test: 1.08±0.09 AU; HI, Pre-test: 1±0.06 AU, Post-test: 1.19±0.10 AU) and COX IV (LO, Pre-test: 1±0.13 AU, Post-test: 1.17±0.13 AU; HI, Pre-test: 1±0.07 AU, Post-test: 1.18±0.10 AU) protein content (see representative blots, Figure 1B). Maximal activity of CS increased in both the LO (Pre-test: 43.8±4.7 µmol/min/g, Post-test: 47.2±5.1 µmol/min/g) and HI (Pre-test: 43.6±4.5 µmol/min/g, Post-test: 49.9±8.8 µmol/min/g) groups resulting in a significant main effect of training (p<0.05; Figure 1C). Maximal activity of βHAD tended to be higher post-training (p = 0.07) in both the LO (Pre-test: 2.3±1.5 µmol/min/g, Post-test: 2.7±1.9 µmol/min/g) and HI (Pre-test: 2.7±0.7 µmol/min/g, Post-test: 3.1±0.4 µmol/min/g) groups (Figure 1C). No group by time interaction effects were observed for any marker of skeletal muscle oxidative capacity.

### Regulators of Mitochondrial Biogenesis

**Figure 2:**
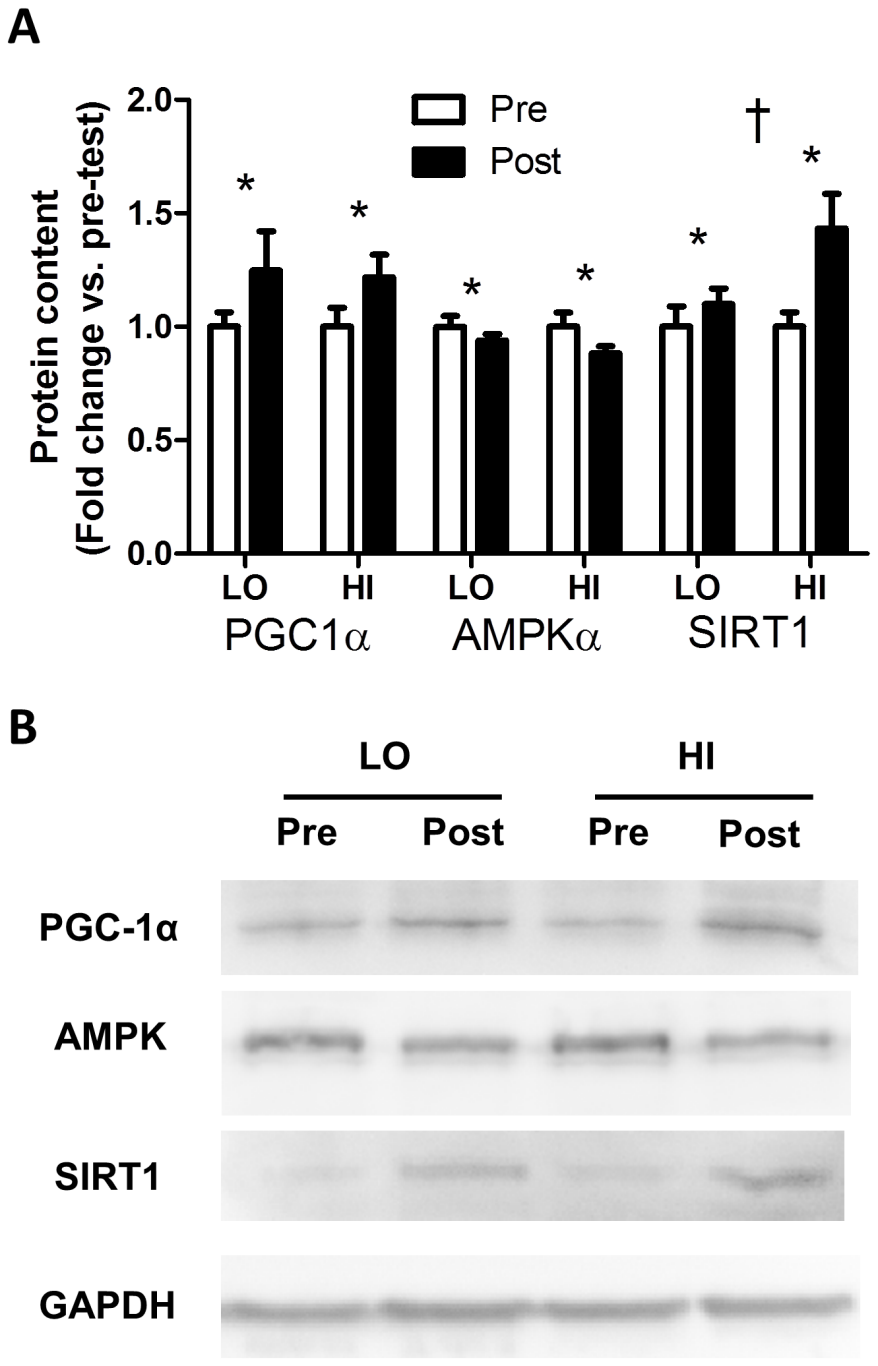
Effects of HI and LO on PGC-1α, AMPK, and SIRT1 protein content. Changes in the protein content of PGC-1α, AMPK and SIRT1 (A). Representative western blots, including loading controls, are also shown (B). * Significant (p<0.05) effect of training. † Significant (p<0.05) interaction.

There was a main effect of training for PGC-1α whole muscle protein content (LO, Pre-test: 1±0.06 AU, Post-test: 1.24±0.17 AU; HI, Pre-test: 1±0.08 AU, Post-test: 1.22±0.09 AU; p<0.05; Figure 2A). A significant effect of training (p<0.05) was also observed for AMPK (LO, Pre-test: 1±0.05 AU, Post-test: 0.94±0.03 AU; HI, Pre-test: 1±0.06 AU, Post-test: 0.88±0.03 AU) and SIRT1 (LO, Pre-test: 1±0.09 AU, Post-test: 1.10±0.07 AU; HI, Pre-test: 1±0.06 AU, Post-test: 1.43±0.15 AU) protein content with a significant (p<0.05) group by time interaction effect observed for SIRT1 (Figure 2A, representative blots Figure 2B).

### VO_2_peak and Submaximal Exercise Performance

**Figure 3:**
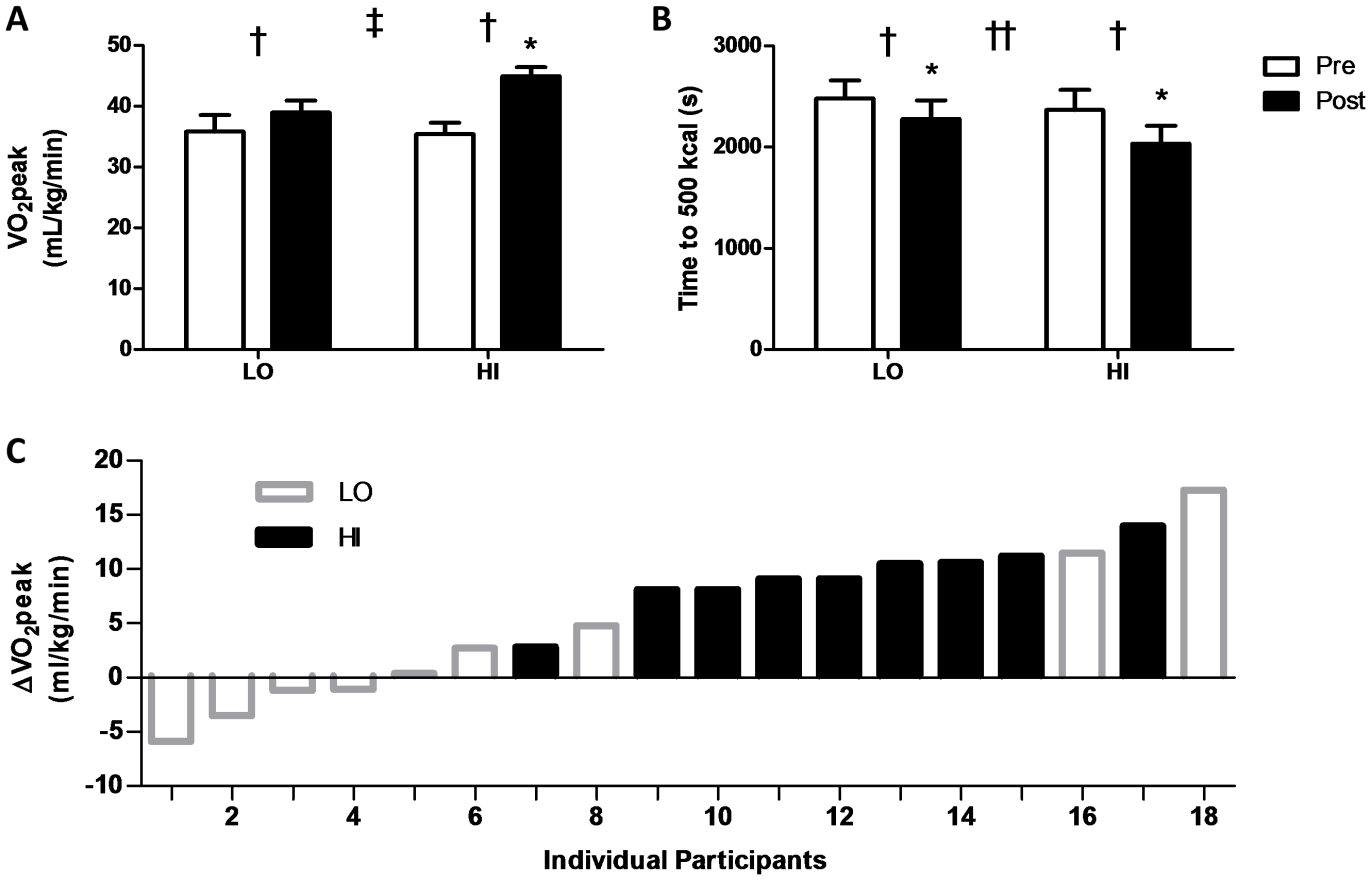
Improvements in VO_2_peak and exercise performance are greater following HI than LO. The mean VO_2_peak (A) and time to 500 kcal (B) for the LO and HI groups are shown. The individual change in VO_2_peak for all participants are also shown (C). *Significant (p<0.05) difference from Pre. † Significant (p<0.05) effect of training. ‡ Significant (p<0.05) interaction. ††Non-significant (p = 0.07) interaction.

**Figure 4:**
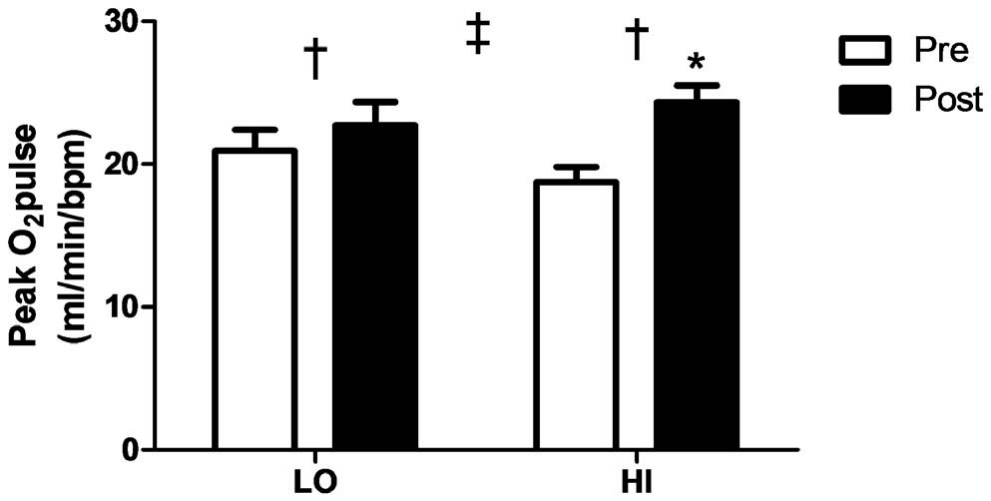
Peak O_2_ pulse increases to a greater extent following HI than LO. *Significant (p<0.05) difference from Pre. † Significant (p<0.05) effect of training. ‡ Significant (p<0.05) interaction.

One participant from the LO group was unable to complete VO_2_peak testing due to intolerability of the apparatus. A significant main effect of training (p<0.001) and a significant group by time interaction effect (p<0.05) were observed for VO_2_peak (Table 1; Figure 3A). Further, only 5 participants in the LO group demonstrated an elevated VO_2_peak following training compared to all 9 participants in the HI group (Figure 3C). A significant main effect of training (p<0.05) was also observed for peak power and peak HR during the ramp protocol (Table 1). A main effect of training (p<0.001) was demonstrated for the time to complete 500 kcal test (Table 1; Figure 3B). The group by time interaction effect did not reach statistical significance (p = 0.07), but indicates a trend towards differing adaptations between groups similar to that observed for VO_2_peak. Significant (p<0.05) effects of training and a group by time interaction effect were observed for peak O_2_ pulse (Table 1; Figure 4).

### Insulin Sensitivity and Inflammatory Markers

No changes in fasting glucose, insulin or HOMA scores were observed in either group. Plasma adiponectin concentrations decreased by 12.9% in the LO group and 19.4% in the HI group with a significant main effect of training observed (p<0.05, Table 2). No effect of training was detected in plasma concentrations of either IL-6 (p = 0.64) or TNFα (p = 0.31) following training.

**Table 2:** Effect of training on plasma pro- and anti-inflammatory markers.

	LO		HI	
	Pre	Post	Pre	Post
**Adiponectin** **(ng/ml)**	81.60±42.32	71.06±28.24†	68.40±25.62	55.14±15.94†
**IL-6 (pg/ml)**	1.74±1.31	1.69±1.46	1.79±0.89	2.27±1.00
**TNFα (pg/ml)**	2.22±1.61	2.07±1.55	1.83±1.03	1.84±0.76

Values are mean ± SD. IL-6, interleukin-6; TNFα, tumor necrosis factor alpha; ng/ml, nanograms per ml; pg/ml, picograms per ml.

† Significant (p<0.05) effect of training.

### Psychological Measures

Acute affect scores were significantly lower (p<0.001) in the HI group throughout the first training session, decreasing an average of 6.9±2.5 points on the Feeling Scale by the end of the 8^th^ interval compared to only 1.4±1.1 points in the LO group. There were no significant (p>0.05) differences in the reports of perceived enjoyment (LO, 6.2±0.9; HI, 6.1±0.8), scheduling self-efficacy (LO, 8.1±2.0; HI 7.9±1.4), or task self-efficacy (LO, 8.8±1.5; HI, 8.4±2.3) between groups following the training intervention. There was also no group effect on the mean reports of intension to implement high intensity exercise (LO, 5.2±1.0; HI, 5.4±1.2, data not shown).

## Discussion

This study sought to determine the impact of HIT dose, specifically the effect of interval intensity and training volume, on skeletal muscle oxidative capacity, aerobic capacity, exercise performance, peak O_2_ pulse, inflammation status, and perceived tolerance. Following a 3-week training intervention in overweight and obese young men: 1) increases in skeletal muscle oxidative capacity were present in both groups and were not different between groups, 2) aerobic capacity and exercise performance were improved in both the LO and HI groups with incremental improvements occurring in an intensity/volume dependent fashion, 3) peak O_2_ pulse increased to a greater extent in the HI group, suggesting that the intensity/volume dependent improvements in VO_2_peak observed following HI are primarily attributable to greater cardiovascular adaptations, 4) markers of systemic inflammation were largely unchanged by either HIT protocol, and 5) despite a more negative affective response during HI intervals, both groups reported similar high levels of enjoyment and self-efficacy.

### Muscle Oxidative Capacity and Mitochondrial Content

Based on previous reports of greater increases in PGC-1α mRNA following acute bouts of higher intensity exercise [18], [19], we hypothesized that mitochondrial content would increase to a greater extent following higher intensities of HIT. Contrary to this hypothesis, there were no statistical differences observed between groups in the changes in either protein content of COX I or COX IV (Figure 1A) or the maximal activities of CS or βHAD (Figure 1C).

The existence of an intensity effect on mitochondrial adaptation has been demonstrated in murine muscle [20]. However, the present results, combined with the typically equivalent adaptations observed between HIT and lower intensity ET [4], [21] question whether this relationship extends to humans. While comparisons between HIT and ET are complicated by differences in both exercise volume (duration and energy expenditure [22]) and potential differences in fiber type recruitment [23], there is currently little evidence available supporting a dose-dependent effect of intensity/volume on mitochondrial adaptations following HIT, or following exercise training in general. It is important to note that for both CS (LO +8%; HI +15%) and COX I (LO +8%; HI +19%) the lack of a statistically significant difference between groups may reflect a lack of statistical power rather than the absence of a difference between interventions. However, while the low statistical power is a limitation of the current study, the lack of significance for CS and COX I based on the present sample, combined with the equivalent changes in βHAD (LO +16%; HI +16%) and COX IV (LO +17%; HI +18%) suggest that reducing both the intensity and volume of HIT may not result in reduced mitochondrial biogenesis. In order to overcome this aforementioned limitation there is a need for future studies examining the impact of exercise intensity on mitochondrial biogenesis to be performed on larger samples than that examined in the present study, and in the bulk of the currently available literature.

The observed increase in PGC-1α following HIT is consistent with previous reports [24], [25], as is the apparent relationship between changes in PGC-1α and changes in oxidative capacity [24], [25]. Interestingly, our findings of similar increases in PGC-1α protein between groups (Figure 2A) suggest that chronic upregulation of this protein is not dependent on interval intensity/volume. This result is not in agreement with recent demonstrations of intensity dependent increases in PGC-1α mRNA following an acute bout of exercise [18], [19], and suggests that either regulation of PGC-1α expression following acute exercise is not as tightly tied to intensity as previously believed or, that intensity dependent changes in RNA do not predict chronic changes in protein content. The mechanisms underlying equivalent changes in PGC-1α protein despite substantial differences in training dose (intensity/volume) require further study.

The observed increase in whole muscle SIRT1 protein content, which appears to be intensity/volume dependent (LO, +9%; HI, +43%; Figure 2A), adds to the discrepant findings surrounding changes in SIRT1 following exercise training [24]–[26]. There is currently extensive controversy in the literature regarding the importance of SIRT1 in skeletal muscle *in vivo*. Specifically, there has been considerable inconsistency in the changes in SIRT1 that accompany exercise mediated changes in mitochondrial content, and results from overexpression/knock out models have not yielded the expected results (please see [27], [28] for a detailed review of this controversy). The present results further highlight the need for future work examining the implications of altered whole muscle SIRT1 in humans, and the role of SIRT1 protein content in determining skeletal muscle mitochondrial content *in vivo*.

### VO_2_peak and Submaximal Exercise Performance

This study represents one of the first attempts to examine the impact of altered interval intensity and volume on aerobic fitness and submaximal exercise performance. Importantly, while intervention with intervals at both 70% and 100% of peak aerobic power improved aerobic capacity and exercise performance, these improvements were greater following HI than LO (Figure 3). These results agree with previous reports demonstrating that improvements in VO_2_peak following steady-state endurance training (ET) occur in an intensity dependent fashion [29]. This apparent intensity effect on increases in VO_2_peak may help explain why improvements in VO_2_peak following HIT are sometimes equivalent [5] or superior [30], [31] to those observed following ET despite the significantly lower exercise volume typically associated with HIT. It is important to note that the lesser aerobic adaptations observed in LO group of the present study may also be attributable to a lower total training volume. At present, we are unable to comment with certainty on the respective contribution of intensity and training volume on the aerobic adaptations observed in the present study. However, given the clinical relevance of VO_2_peak [32], and despite the possible psychological [7] and safety [33] concerns associated with high intensity exercise, these results highlight the importance of continuing to promote high intensity and volume when HIT is prescribed to overweight and obese populations.

### Cardiovascular Adaptation to HIT

Classically, changes in VO_2_peak have been linked to improvements in stroke volume (SV), cardiac output (CO), and the ability of the cardiovascular systems ability to deliver O_2_ [34]. Consistent with this view, several recent reports have demonstrated concomitant increases in SV and VO_2_peak following training [35] and intensity dependent increases in SV [31]. While we did not measure SV or CO in the current study, the observed increase in peak O_2_ pulse (Figure 4) suggests that the greater improvement in VO_2_peak observed in the HI group was the result of greater cardiovascular adaptations. Coupled with the similar increases in skeletal muscle oxidative capacity in both groups (Figure 1), previous observations that oxidative capacity and capillary density increase in concert [36], and reports that changes in peak O_2_ pulse are strongly correlated with increases in SV [37], [38], our data seem to suggest that increases in SV following HIT are dependent on both intensity and training volume. While this is an attractive explanation for our results, further studies confirming an intensity dependent increase in SV following interval training, the mechanism(s) underlying this effect, and the minimal intensity required to increase cardiovascular function are needed.

### Systemic Inflammation

Chronic low-grade inflammation is a hallmark of obesity and may contribute to a number of diseases such as type 2 diabetes and metabolic syndrome [39]. While previous exercise interventions have shown reductions in markers of systemic inflammation [40], we did not detect any decreases in the proinflammatory markers IL-6 or TNFα following HIT (Table 2). The lack of change observed in these two inflammatory factors may indicate that improvements in inflammation require a longer period of regular exercise than 3 weeks [3] or that the population examined in the present study (young overweight/obese adults) did not exhibit low-grade inflammation prior to our intervention. In support of the latter contention, the resting values for both TNFα (∼2 pg/ml) and IL-6 (∼1.75 pg/ml) are below the values typically reported in individuals with diagnosed, or elevated risk for metabolic disease (TNFα >4 pg/ml; IL-6, ∼2 pg/ml) [3], [40], [41]. The observed decrease in adiponectin following HIT in both groups (Table 2) is in agreement with previous work [42] and may reflect slight increases in cytokine release following each training bout modestly impairing adiponectin release [43]. However, at present the influences of intensity, volume, duration and modality of exercise training on adiponectin levels, along with the implications associated with altered resting adiponectin levels remains unclear and remains an important area of future study.

### Interval Intensity and Tolerability of HIT

In accordance with Ekkekakis’ Dual-Mode Model (DMM) [7], the affective response to the intervals was significantly lower in participants performing the HI protocol. DMM proposes that affect – how pleasurable exercise is perceived to be – declines considerably as the intensity of exercise increases. Given that affect assessed during an exercise bout predicts engagement in that exercise behaviour up to 12 months afterwards [44], these findings suggest that intervals performed at high intensities may not be adhered to. Interestingly, and in contrast to the affect data of the current study, participants reported equally high ratings of enjoyment in both exercise intensity groups. Further, participants in both groups demonstrated high confidence to successfully complete high-intensity intervals and schedule high-intensity interval exercise into their weekly routine. These findings support preliminary reports of enjoyment of high-intensity interval exercise [6]. The finding that self-efficacy was equally high in both conditions suggests that participants perceived HIT as manageable and were confident that they could schedule such activity into their lives on a regular basis. Future research examining if these perceptions actually translate to increases in adherence to HIT is required.

### Practical Implications and Future Directions

Our results suggest that not all health benefits are lost when HIT is performed at lower, less demanding intensities. However, the greater improvement in aerobic capacity and exercise performance observed in the HI group indicate that training at higher intensities, with higher training volumes augments the training response. Interestingly, the similar responses from participants on measures of exercise enjoyment and scheduling and task self-efficacy despite significantly lower acute affect in the higher intensities suggests that prescription of higher intensities of training for sedentary populations may not preclude adherence and may support the validity of high intensity training as an exercise strategy in the general population. Despite the similar mitochondrial adaptations observed, our results suggest that intensity and volume should continue to be stressed should health practitioners choose to prescribe HIT to overweight/obese/diseased populations.

### Summary

In summary, we have examined the impact of low intensity/low volume and high intensity/high volume interval training in overweight and obese young adults. Our results indicate that improvements in aerobic capacity and exercise performance are intensity/volume dependent but changes in markers of skeletal muscle oxidative capacity are not. These results, combined with a greater change in O_2_ pulse in the high intensity group suggest that the additional improvements in aerobic capacity are a result of a greater cardiovascular adaptation, possibly through enhanced stroke volume. Further research concerning the impact of interval intensity on mechanisms underlying the adaptations to HIT would be beneficial for optimization of HIT protocols and exercise prescription.
